# Plasmonic and photonic scattering and near fields of nanoparticles

**DOI:** 10.1186/1556-276X-9-50

**Published:** 2014-01-29

**Authors:** Martina Schmid, Patrick Andrae, Phillip Manley

**Affiliations:** 1 Helmholtz-Zentrum Berlin für Materialien und Energie, Nanooptical Concepts for PV, Hahn-Meitner-Platz 1, 14109 Berlin, Germany

**Keywords:** Nanoparticles, Plasmonics, Photonics, Scattering, Near field, Mie theory, FEM simulations, Solar cells, 42.70.-a, 78.67.Bf, 73.20.Mf

## Abstract

We theoretically compare the scattering and near field of nanoparticles from different types of materials, each characterized by specific optical properties that determine the interaction with light: metals with their free charge carriers giving rise to plasmon resonances, dielectrics showing zero absorption in wide wavelength ranges, and semiconductors combining the two beforehand mentioned properties plus a band gap. Our simulations are based on Mie theory and on full 3D calculations of Maxwell’s equations with the finite element method. Scattering and absorption cross sections, their division into the different order electric and magnetic modes, electromagnetic near field distributions around the nanoparticles at various wavelengths as well as angular distributions of the scattered light were investigated. The combined information from these calculations will give guidelines for choosing adequate nanoparticles when aiming at certain scattering properties. With a special focus on the integration into thin film solar cells, we will evaluate our results.

## Background

The study of light scattering from small particles goes back for more than a hundred years, as shown by the early theory by Mie in 1908 [1], but applications have been known since much longer, see for example the Lycurgus cup [2]. Currently, nanoparticles find widespread applications in elaborate technologies - and they also require elaborate selection and tuning for each of the individual applications. The specific scattering of nanoparticles was shown to be beneficial for enhanced outcoupling from LEDs [3], in nano-waveguides [4] or nano-antennas [5]. The enhanced near fields are exploited, e.g., in Raman spectroscopy [6], near field optical microscopy [7], or biosensing [8].

Another promising application for plasmonic and photonic nanoparticles is in photovoltaic devices for absorption enhancement. Both metallic and dielectric nanoparticles have been used for this purpose: Ag nanoparticles in Si solar cell [9,10], Au and SiO_2_ on Si [11], SiO_2_ on Si [12], Ag on GaAs [13], Ag in organic solar cells [14], Ag in dye-sensitized solar cells [15], etc. There appears to have been a strong focus on Ag nanoparticles, yet also SiO_2_ nanoparticles are growing in interest. According to [16,17], several mechanisms have to be taken into account when considering plasmonic nanoparticles for solar cell applications: enhanced near fields, high (angle) scattering, and in the case of regular arrangements, coupling into guided modes. Also, the dielectric nanoparticles come with their specific promises for expected enhancement [18,19]. But which nanoparticle material will provide the most efficient light coupling?

In a solar cell, the objectives for nanoparticle application are as follows: in ultra-thin or low-absorbing photovoltaic materials, plasmonic and photonic nanoparticles are expected to enhance the absorption. This can be achieved by various mechanisms which ideally can be combined or for which the most promising one needs to be identified. Firstly, nanoparticles may be able to locally concentrate light into their vicinity, i.e., generate a near-field enhancement, which then can lead to enhanced absorption in a surrounding medium. Secondly, they scatter light and therefore are able to redirect the initially incident light for preferential scattering into the solar cell, similar to traditional anti-reflection coatings or back reflectors. Thirdly, the scattered light is ideally scattered into modes that are otherwise subject to total reflection (being related to a high angular scattering distribution) which leads to light trapping in a thin layer. Finally, strong fields at interfaces can also lead to leaky modes enhancing the absorption in the vicinity similarly to the near fields.

With the aim of judging which type of material is the most promising one for the desired absorption enhancement, we compare the absorption and scattering behavior of different materials, each of which is characterized by a particular refractive index. The task is to find how the optical properties will influence the plasmonic/photonic scattering behavior and how we need to tune according parameters. We compare metals and dielectrics but will also address semiconductors, since for example the scattering of silicon nanoparticles has started to attract interest [20].

## Methods

### Mie theory

We calculate the elastic interaction of an electromagnetic wave with a homogenous spherical particle using the Mie solution to Maxwell’s equations. The Mie theory gives the scattered external (scattering, extinction) and internal field of the particle (absorption, field penetration inside the sphere). The matrix form can be used to show the relation between incident (subscript *I*) and scattered (subscript *S*) fields:

(1) $$\left(\begin{array}{c} {E_{\left|\right|}}_{S}\\ E_{⊥S} \end{array}\right)=\frac{e^{i\frac{2\pi }{\lambda }\mathrm{res}}}{-i\frac{2\pi }{\lambda }\mathrm{res}}\;\left(\begin{array}{cc} S_{2} & S_{3}\\ S_{4} & S_{1} \end{array}\right)\;\left(\begin{array}{c} {E_{\left|\right|}}_{I}\\ E_{⊥I} \end{array}\right)$$

Where *res* is the resulting vector of the far field, *S* is the amplitude scattering matrix, and *λ* is the wavelength of the incident light with the electromagnetic wave components *E*_∥__*I*_ and *E*_⊥__*I*_. The scattering amplitudes can be solved for a sphere with *S*_3_ = *S*_4_ = 0. However, the result of the scattering amplitudes *S*_1_ and *S*_2_ will still depend on the scattering angle and azimuthal angle. For the calculation in the Mie simulation of nanoparticles with variable radius, we concentrate on calculating the cross section with the Mie coefficients, which will no longer depend on the scattering angles. First, we calculate the Mie coefficients for the external field in an infinite and homogenous medium [21]:

(2) $$a_{l}=\frac{ñ\;\left(\lambda \right)\psi_{l}\left(ñ\;\left(\lambda \right)x\right){\psi^{'}}_{l}\left(x\right)-\psi_{l}\left(x\right)\psi {'}_{l}\left(ñ\;\left(\lambda \right)x\right)}{ñ\;\left(\lambda \right)\psi_{l}\left(ñ\;\left(\lambda \right)x\right){\xi^{'}}_{l}\left(x\right)-\xi_{l}\left(x\right){\psi^{'}}_{l}\left(ñ\;\left(\lambda \right)x\right)}$$

(3) $$b_{l}=\frac{\psi_{l}\left(ñ\;\left(\lambda \right)x\right){\psi^{'}}_{l}\left(x\right)-ñ\;\left(\lambda \right)\psi_{l}\left(x\right)\psi {'}_{l}\left(ñ\;\left(\lambda \right)x\right)}{\psi_{l}\left(ñ\;\left(\lambda \right)x\right){\xi^{'}}_{l}\left(x\right)-ñ\left(\lambda \right)\xi_{l}\left(x\right){\psi^{'}}_{l}\left(ñ\;\left(\lambda \right)x\right)}$$

Where *l* is the mode, *x* is the size parameter $x=\frac{2\pi }{\lambda }r$, and *ñ* is the complex reflective index. To simplify the formulas for calculation, the Riccati-Bessel functions *ψ*_*l*_(*p*) and *ξ*_*l*_(*p*) are used. We can calculate the scattered field by using the boundary conditions and adding up the resulting wave vectors of the particle scattering leading to the scattering cross section *C*_sca_ and the extinction cross section *C*_ext_:

(4) $$C_{\mathrm{sca}}=\frac{\lambda^{2}}{2\pi }\;\sum_{l=1}^{\infty }\left(2l+1\right)\left({\left|a_{l}\right|}^{2}+{\left|b_{l}\right|}^{2}\right)$$

(5) Cext=λ22π∑l=1∞2l+1Real+bl

The absorption cross section *C*_abs_ results as

(6) $$C_{\mathrm{abs}}=C_{\mathrm{ext}}-C_{\mathrm{sca}}$$

The normalized cross sections *Q* - which we will show in the following - are calculated by dividing *C* through the particle area *πr*^2^. The different modes and the separation of the electric and magnetic field is done by the individual calculation of *a*_*l*_ and *b*_*l*_ with *l* for any relevant number (e.g., 1, 2, 3, 4,…).

The scattering efficiency is defined as

(7) $$Q_{\mathrm{eff}}=\frac{Q_{\mathrm{sca}}}{Q_{\mathrm{sca}}+Q_{\mathrm{abs}}}$$

### 3D FEM calculations

We solve Maxwell’s equations in full 3D with the finite element method (FEM) using the software package JCMwave, Berlin, Germany [22]. The FEM is a variational method whereby a partial differential equation is solved by dividing up the entire simulation domain into small elements. Each element provides local solutions which, when added together, form a complete solution over the entire domain. Due to the inherently localized nature of the method, different regions of space can be modeled with different accuracy. This allows demanding regions like metallic interfaces to be calculated with a high accuracy without compromising on total computation time.

The time harmonic ansatz along with the assumptions of linear, isotropic media and no free charges or currents allows Maxwell’s equations to be written as a curl equation:

(8) $$\frac{1}{\epsilon }\nabla \times \frac{1}{\mu }\nabla \times E\;-\;\omega^{2}E=0$$

Where *ϵ* and *μ* are the permittivity and the permeability of the medium respectively, ***E*** is the electric field vector, and *ω* is the frequency of the electromagnetic radiation. This equation can be solved numerically by discretization of the curl operator (∇×) using the finite element method. After the discretization, a linear system of equations needs to be solved to calculate the field scattered by the geometry in question. During our calculations, the finite element degree and grid discretization were refined to ensure a convergence in the scattering and absorption cross sections to the 0.01 level.

For the calculation of normalized scattering and absorption cross sections, the Poynting flux of the scattered field through the exterior domain and the net total flux into the absorbing medium were used. The normalized cross section is then:

(9) $$Q=\frac{\Phi }{\Phi_{I}}/\frac{C_{N.P.}}{C_{C.D.}}$$

Where *Φ* is the scattered or absorbed flux, *Φ*_*I*_ is the incident flux, and *C*_N.P*.*_ and *C*_C.D*.*_ are the cross-sectional area of the nanoparticle and computational domain, respectively. The calculation of the angular far field spectrum is achieved by an evaluation of the Rayleigh-Sommerfeld diffraction integral.

In our calculations, Mie theory was mainly used for extraction of scattering and absorption cross sections, their division into the different order electric and magnetic modes and their representation as maps of wavelength and nanoparticle radius. The electromagnetic near fields and the angular distributions of scattered light were preferentially calculated with 3D FEM simulations. Whereas Mie theory is a fast calculation method, it cannot handle nanoparticles at an interface which we will address in our last chapter. The comparison of the two calculation approaches for the simple case of a nanoparticle in vacuum (air) gives us confidence about the conformity of the two methods where possible. If not stated otherwise, a spherical nanoparticle in air is investigated and cross sections are always the normalized values.

### Dielectric function of materials

For the above mentioned calculation methods along with the particular geometry, the optical constants of the materials, i.e., the dielectric functions, are the fundamental input parameters. Therefore, we now bring together the essentials of describing the dielectric function of a material which we will use in the following.

The dielectric function *∈* = *∈*_1_ + *i∈*_2_ relates to the refractive index *ñ* = *n* + *ik* as

(10) $$\epsilon ={ñ}^{2}$$

The dielectric function of a material strongly depends on its electronic states: metals are dominated by free electrons whereas dielectrics have no free movable charges and semiconductors are characterized by a band gap plus possibly free charge carriers. The corresponding dielectric functions are often times described by models of which the most common ones are summarized below:

### Metals - Drude formula

(11) $$\epsilon =1-\frac{\omega_{p}^{2}}{\omega^{2}+\mathrm{i\omega \gamma}}$$

With the damping *γ* and the plasma frequency *ω*_*P*_ related to the free charge carrier concentration *n*_*e*_ and the effective mass m^*^ by ℏ

(12) $$\omega_{p}=\sqrt{\frac{n_{e}e^{2}}{m^{*}\epsilon_{0}}}=E_{p}/\hbar$$

Whereas the plasma frequency relates to a property of a bulk material, for a spherical nanoparticle with radius *r* made from a material that can be described by the Drude formula, the resonance conditions for particle plasmons given by *∈* = −2 may be fulfilled. This condition results from the polarizability *α* which is derived for small particles [21] as

(13) $$\alpha =4\pi \epsilon_{0}r^{3}\frac{\epsilon -1}{\epsilon +2}$$

Metals may also show significant interband transitions and related absorption which can be described by a Lorentz oscillator compare also the semiconductors.

### Dielectrics - Cauchy equations

(14) $$n^{2}=1+\frac{B_{1}\lambda^{2}}{\lambda^{2}-C_{1}}+\frac{B_{2}\lambda^{2}}{\lambda^{2}-C_{2}}+\frac{B_{3}\lambda^{2}}{\lambda^{2}-C_{3}}$$

With the Sellmeier coefficients *B*_1, 2, 3_ and *C*_1, 2, 3_. The Cauchy equation can be approximated by a constant refractive index value for longer wavelengths.

### Semiconductors - Tauc-Lorentz model

Combine the Tauc joint density of states with the Lorentz oscillator model for *∈*_2_:

(15) $$\epsilon_{2}=\left\{\begin{array}{r} \frac{AE_{0}C{\left(E-E_{g}\right)}^{2}}{{\left(E^{2}-E_{0}^{2}\right)}^{2}+C^{2}E^{2}}\cdot \frac{1}{E}\;,\;E>E_{g}\\ 0\;,\;E<E_{g} \end{array}\right)$$

and *∈*_1_ is defined according to the Kramers-Kronig relation

(16) $$\epsilon_{1}\left(E\right)=\epsilon_{1,\infty }+\frac{2}{\pi }P\int_{E_{g}}^{\;\infty }\frac{\zeta \epsilon_{2}\left(E\right)}{\zeta^{2}-E^{2}}\mathrm{d\zeta}$$

For the presence of significant free charge carriers in the semiconductor, the Tauc-Lorentz model can be combined with the Drude formula.

**Figure 1:**
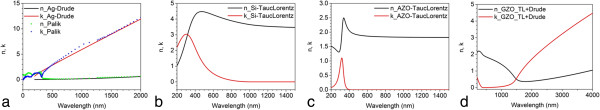
**Refractive index (n,k) of the materials used in the calculations. (a)** Ag with Drude fit, **(b)** a-Si with Tauc-Lorentz fit, **(c)** AZO with Tauc-Lorentz fit, and **(d)** GZO with combined Tauc-Lorentz and Drude fit; fitting parameters according to Table 1.

For the materials used in the following calculations, we give the parameters used to fit the dielectric function with the models above in Table 1 and show the refractive index (n,k) in Figure 1.

**Table 1:** Fitting paramaters for the materials used in the calculations

	***A *** **(eV)**	***C *** **(eV)**	***E***_**0 **_**(eV)**	***E***_**g **_**(eV)**	∈ _**1,** ***∞***_	***E***_**p **_**(eV)**	***γ *** **(eV)**
Ag (fitting Palik [23])	-	-	-	-	-	7.44	0.062
Dielectric (const)	-	-	-	-	4	-	-
a-Si (Jellsion [24,25])	122	2.54	3.45	1.20	1.15	-	-
AZO (Gao [26])	42.8	0.476	3.79	2.951	2.69	-	-
GZO (Fujiwara [27])	139.4	15.0	7.3	3.14	1	1.593	0.130

Fitting parameters according to Equations 15 and 16 (*A*, *C*, *E*_0_, *E*_*g*_, ∈ _1,*∞*_) and Equations 11 and 12 (*E*_*p*_, *γ*) for the materials used in the calculations.

## Results and discussion

We start with investigating the scattering and near fields of metallic nanoparticles and later contrast them to those from dielectric particles. These considerations will further lead us to address nanoparticles made from semiconducting materials. To finally evaluate the efficiency of the nanoparticles’ scattering for light trapping purposes, we will address the angular distribution of the scattered light including the consideration of a substrate.

### Metals

**Figure 2:**
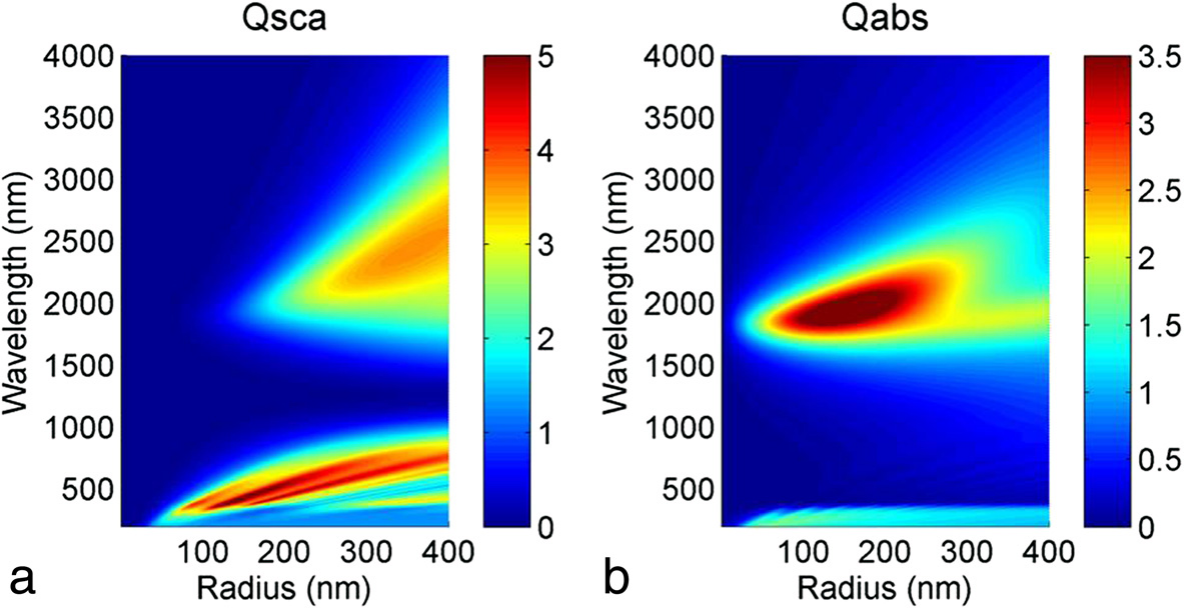
**Scattering maps for metallic nanoparticles. (a)** Scattering cross section and **(b)** scattering efficiency of a spherical Ag nanoparticle with refractive index data obtained from a Drude fit to data from Palik.

The dielectric function of a metal being characterized by the free electrons can, in wide ranges, be described by the Drude formula (see Equation 11). As a metal, Ag was chosen, which is the most popular material for plasmonic application since it has a low absorption in the visible region. A fit to the Drude equation with plasma frequency as given in Table 1 results in a good approximation of Ag data from Palik [23] in the wavelength range above 300 nm; below interband transitions exist which cannot be reproduced with this model (compare Figure 1a). In Figure 2, the scattering cross section *Q*_sca_ and the scattering efficiency *Q*_eff_ are shown in subfigures a and b, respectively, for a Drude-fitted Ag spherical nanoparticle in air. These maps of scattering efficiency as a function of wavelength and particle radius can quickly be calculated based on Mie theory. They allow the estimation of the required particle size for most effectively exploiting the scattering while having a low parasitic absorption and for tuning the resonance frequency to the desired wavelength range. From Figure 2, we can see that nanoparticles with a radius of <50 nm are subject to strong absorption, whereas nanoparticles with *r* = 50 nm are already dominated by scattering. The related resonance wavelengths however appear at *λ* < 500 nm. In terms of the application to devices which mainly work in the visible range of light, a shift of the main resonance to *λ* approximately 700 nm is desirable and can be achieved by choosing bigger nanoparticles - *r* = 120 nm appears a good choice judging from the maps in Figure 2.

**Figure 3:**
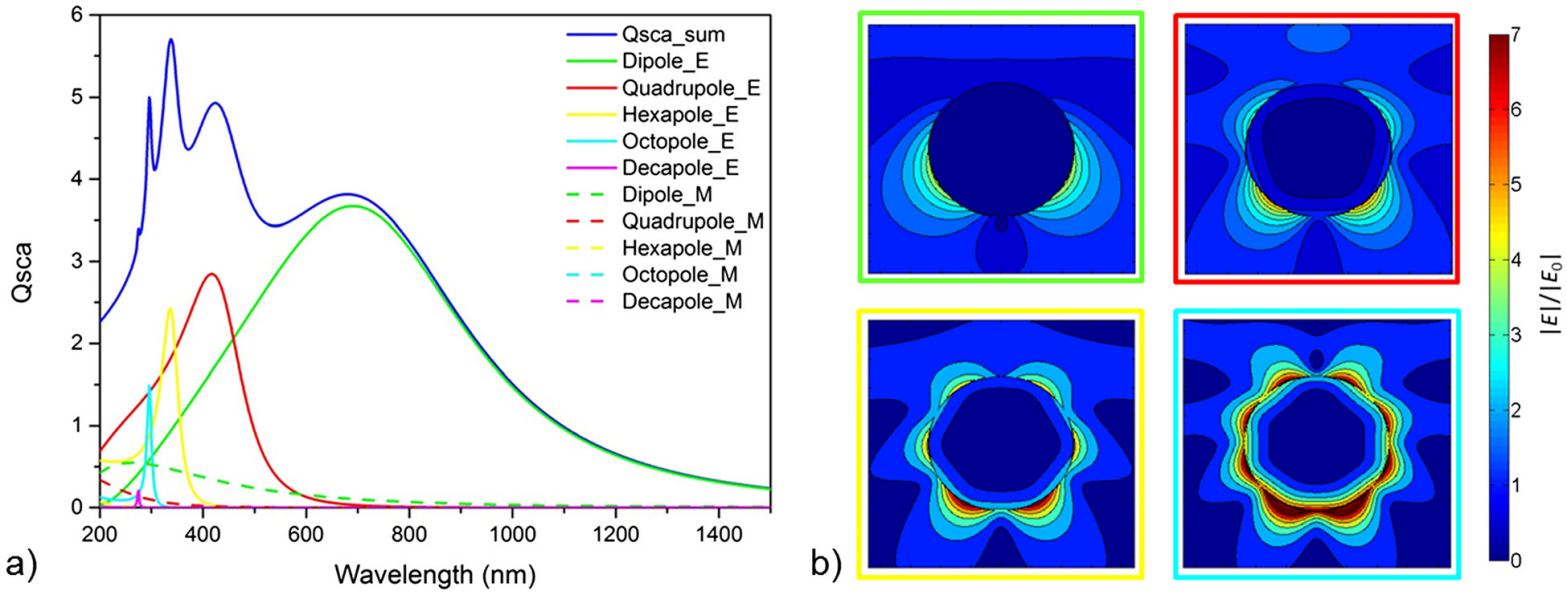
**Scattering and near fields of a metallic nanoparticle. (a)** Scattering cross section of a 120-nm radius Ag nanoparticle with dielectric function according to a Drude fit; sum and allocation to different order and electromagnetic (E/M) modes. **(b)** Near field distribution of the electromagnetic field around the nanoparticle for the dipole, the quadrupole, the hexapole, and the octopole electric mode at wavelengths of 688, 426, 340, and 298 nm, respectively, which correspond to the maxima in scattering (incident light from the top).

Figure 3a shows the according scattering cross section of a 120-nm radius nanoparticle from Ag with dielectric function fitted according to the Drude model. The sum as well as the division into the individual order modes is given. The main resonance at *λ* approximately 700 nm can be attributed to the dipole electric mode, the dominant peaks at shorter wavelengths related to the quadrupole, the hexapole, and the octopole electric mode. We want to note that for the metallic nanoparticles, the resonance peaks result from maxima of the electric modes. Magnetic modes only appear at shorter wavelengths and are much less pronounced. Comparing the scattering to the absorption cross section (see Additional file 1: Figure S1), the lower order modes, i.e., especially the dipole mode, are more favorable for efficient scattering. The near field distributions of the electromagnetic field around the nanoparticle are given in Figure 3b at the peak wavelengths of the dominant electric modes. Since the nanoparticle investigated is of metallic nature, we find no strong electromagnetic field inside the particle where the free charge carriers can compensate local fields. However, the metal fulfills the particle plasmon resonance condition (see Equation 13), and the related plasmonic collective oscillations of the electrons cause strong electromagnetic fields to build up around the surface of the nanoparticle which are characterized by knots according to the respective order. A slightly stronger electromagnetic field in the forward direction is the result of interference with the incident light.

### Dielectrics

Dielectrics show an imaginary part of the refractive index which is zero, i.e., no absorption, which makes them favorable to be used as the material for scattering nanoparticles. The main question is whether these dielectric nanoparticles can give scattering cross sections comparable to the ones of metallic nanoparticles. The refractive index of a typical dielectric is often times described with a Cauchy model, yet since it is constant over a wide wavelength range, we approximate it with *n* = const (=2 here) and *k* = 0. We choose *n* = 2 since the value is a compromise for the most popular oxides SiO_2_ (*n* approximately 1.5) and TiO_2_ (*n* approximately 2.5) or also Al_2_O_3_ (*n* approximately 1.7) and ZrO_2_ (*n* approximately 2.2). Si_3_N_4_ would be the example with the direct value *n* approximately 2 in the wavelength range above 400 nm.

**Figure 4:**
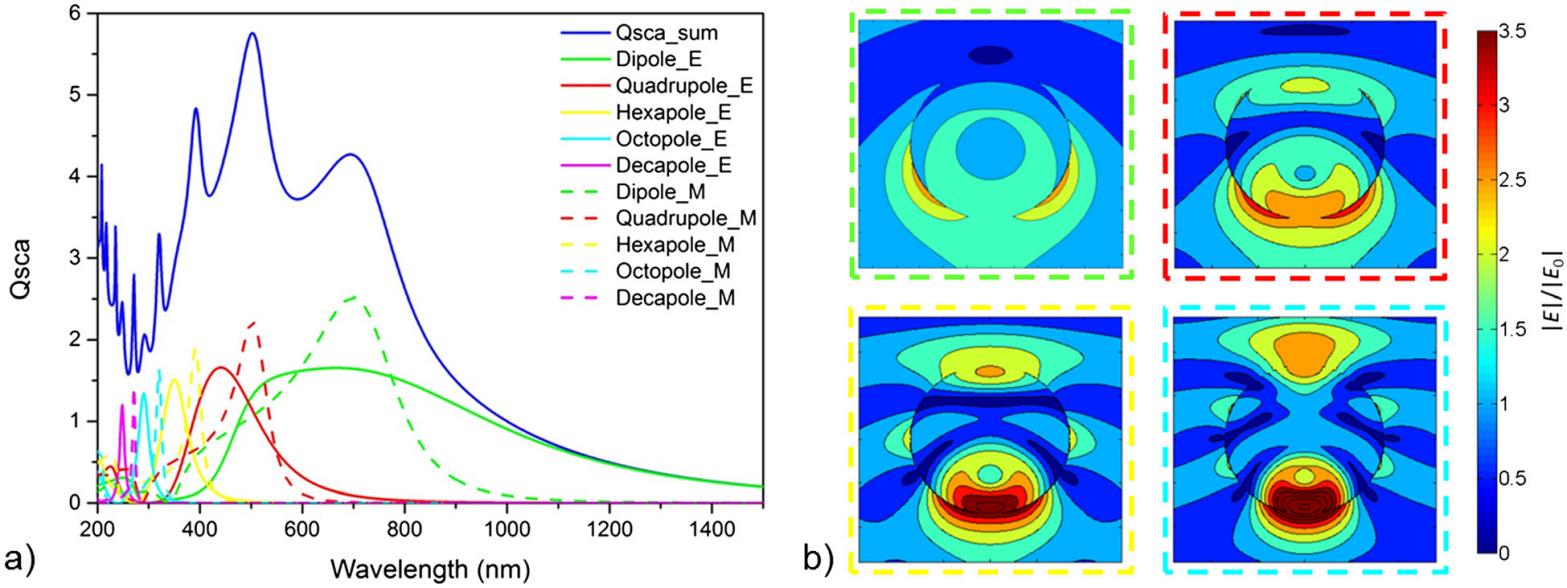
**Scattering and near fields of a dielectric nanoparticle. (a)** Scattering cross section of a 170-nm radius nanoparticle with refractive index *n* = 2 and *k* = 0; sum and allocation to different order and electromagnetic (E/M) modes. **(b)** Near field distribution of the electromagnetic field around the nanoparticle for the dipole, the quadrupole, the hexapole, and the octopole magnetic mode at wavelengths of 700, 502, 392, and 322 nm, respectively, which correspond to the maxima in scattering (incident light from the top).

Scattering cross section maps (the absorption cross sections always being zero) again give guidelines for an adequate radius in order to obtain the main scattering resonance at *λ* approximately 700 nm (see Additional file 2: Figure S2). This requirement is fulfilled for the dielectric nanoparticle (in air) with *n* = 2, *k* = 0 for a radius of 170 nm which is distinctly larger than in the case of metallic nanoparticles (*r* = 120 nm). Figure 4a represents the total scattering cross section with the main resonance around 700 nm together with the division into the different order electromagnetic modes which are manifold for this medium-sized nanoparticle. As Figure 4a shows, the magnetic modes dominate the peaks of the scattering cross section and the electric modes contribute in the form of a broader background. The maximum scattering cross section reaches a value of nearly 6 which is the same as for the 120-nm radius Drude-fitted Ag nanoparticle. From this point of view, the dielectric nanoparticles appear to perform equally well or, considering the zero absorption, even better than the metallic ones. Looking at the near fields of the dominant resonance modes (Figure 4b), however reveals distinct differences: the magnetic modes of the dielectric nanoparticles appear to localize the electromagnetic field inside the particle and the direction of light extraction seems to be preferential to the direct forward direction, i.e., the dielectric nanoparticle appears like a lens. There is a strong near field in this direction in contrast to the remaining surface of the nanoparticle. We will come back to a detailed comparison of the angular distributions of the scattered light in a later section. Here, we only record that dielectric nanoparticles are characterized by a strong scattering, yet not by a pronounced near field enhancement around the particle.

### Semiconductors

After having seen both the benefits of the metallic as well as of the dielectric nanoparticles, we move on to considering nanoparticles of semiconductor material which might combine the two particular properties of free charge carriers and an area of approximately zero absorption. In the case of a semiconductor, furthermore, its band gap needs to be considered which can be achieved using the Tauc-Lorentz combined density of states and an oscillator model.

In our investigations, we address three different semiconductors: amorphous silicon (a-Si), Al-doped ZnO (AZO), and Ga-doped ZnO (GZO). The refractive index data was fitted using parameters from [24,25] for a-Si, from [26] for AZO, and from [27] for GZO, see Table 1. Only the latter one has a significant free charge carrier concentration according to the parameters used here, which leads to a pronounced plasmon resonance; the dielectric function of a-Si and AZO is simply characterized by the band gap and the constant refractive index at longer wavelengths, see also Figure 1b,c,d.

**Figure 5:**
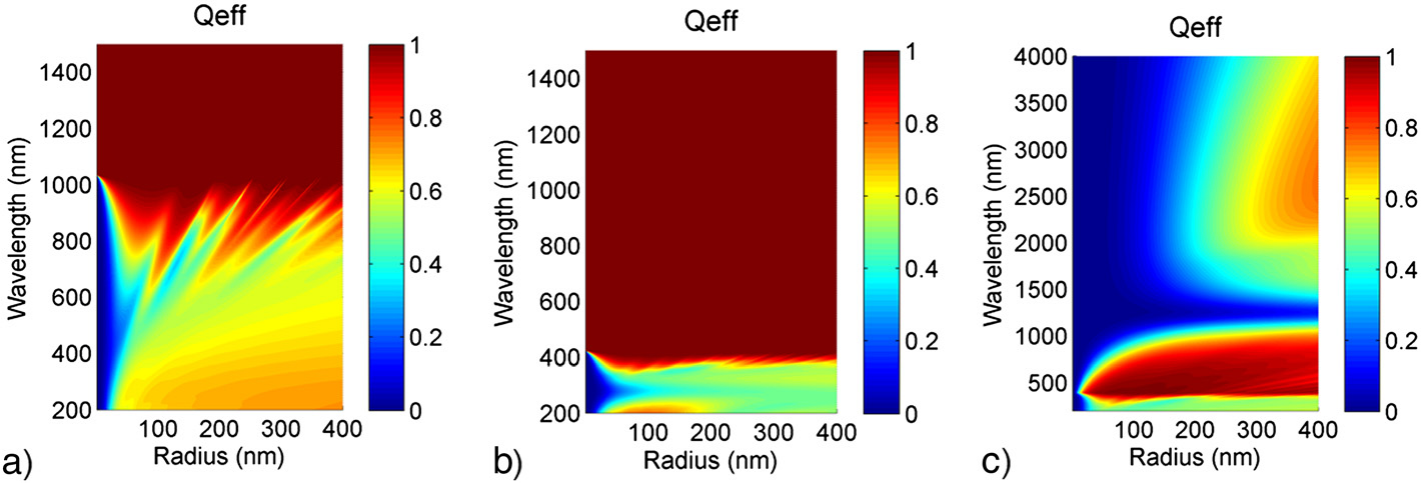
**Maps of scattering efficiency for semiconductor nanoparticles.** Spherical particle made from **(a)** a-Si, **(b)** AZO, and **(c)** GZO with refractive indices fitted with parameters from [24,25], [26], and [27], respectively (note the different wavelength range in **(c)**).

Figure 5 compares the scattering efficiencies for spherical nanoparticles (in air) from the three semiconductors which are characterized by a band gap around 800 nm (for a-Si) and 400 nm (for AZO and GZO). For wavelengths below the band gap (i.e., in terms of energy above), the absorption is dominant, and thus scattering can only be exploited for wavelengths well beyond the band gap. Since this is the case above 1,000 nm only for the a-Si nanoparticles, they cannot be expected to perform well in a device operating in the visible wavelength range. The band gap has to be chosen as low (in wavelengths, but high in energy) as possible. For AZO, the scattering efficiency is 1 for wavelengths larger than the band gap at around 400 nm making it comparable to a dielectric. This is not surprising since low-doped semiconducting materials far away from a specific resonance will show dielectric-like behavior. Comparing a dielectric nanoparticle to one made of a low-doped semiconductor, the latter loses in terms of scattering efficiency since it shows parasitic absorption below the band gap.

**Figure 6:**
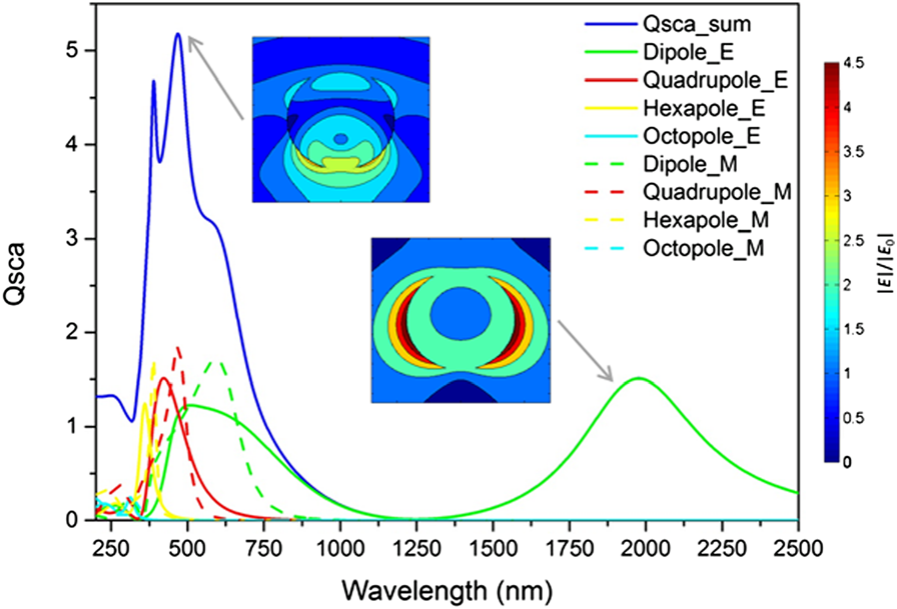
**Scattering and near fields of a semiconductor nanoparticle.** Scattering cross section of a 170 nm radius nanoparticle from GZO (refractive index data fitted with parameters from [27]) and near field distribution of the electromagnetic field around the nanoparticle for the quadrupole magnetic mode at 468 nm and the dipole electric mode at 1,978 nm as insets (incident light from the top.

For the highly doped semiconductor, the situation is slightly different. Also here, parasitic absorption dominates for wavelengths below the band gap. But additionally, the free charge carriers of the highly doped semiconductor lead to further parasitic absorption in the wavelength range where they become dominant, compare Figure 5c (and also see the Additional file 3: Figure S3 for the individual absorption and scattering cross sections). Yet, they also give rise to a plasmonic resonance since the according requirements for the refractive index (*∈*_1_ = −2) can be fulfilled. For GZO, the conditions are met at *λ* approximately 2,000 nm so that a further resonance occurs here. This peak can be attributed to the dipole electric mode as shown in Figure 6 where the sum of the scattering cross section for an *r* = 170 nm GZO nanoparticle is depicted together with the different order electric and magnetic modes. Going from short to long wavelengths, we first see that the band gap and related absorption dominates; then, the constant refractive index gives rise to a dielectric-like scattering behavior with dominant magnetic modes and finally the free charge carriers lead to a plasmon resonance in the infrared. The near field pictures in the inset reveal the typical electromagnetic field distribution of a dielectric nanoparticle for wavelengths up to 600 nm and one commonly seen in metallic nanoparticles at *λ* approximately 2,000 nm. The dielectric modes are virtually identical to the ones shown in Figure 4b; the metal-like mode however no longer occurs as pronounced as in Figure 3b.

The finding for the GZO nanoparticle of low pronounced plasmonic near field modes together with the fact that a plasmon resonance at *λ* = 2,000 nm cannot be exploited when working in the visible regime suggests that we should tune the plasma frequency of the semiconductor such that we obtain a plasmon resonance in the visible. Yet, this would lead us back to the case of a metal described by the Drude formula, so that we once again end up with a trade-off between metallic and dielectric scattering properties.

### Angular scattering distribution and substrate

To further judge whether metallic or dielectric nanoparticles are performing better for light trapping purpose, we now address, in addition to the scattering cross sections and the electromagnetic near field distributions, the angular distribution of the scattered light.

**Figure 7:**
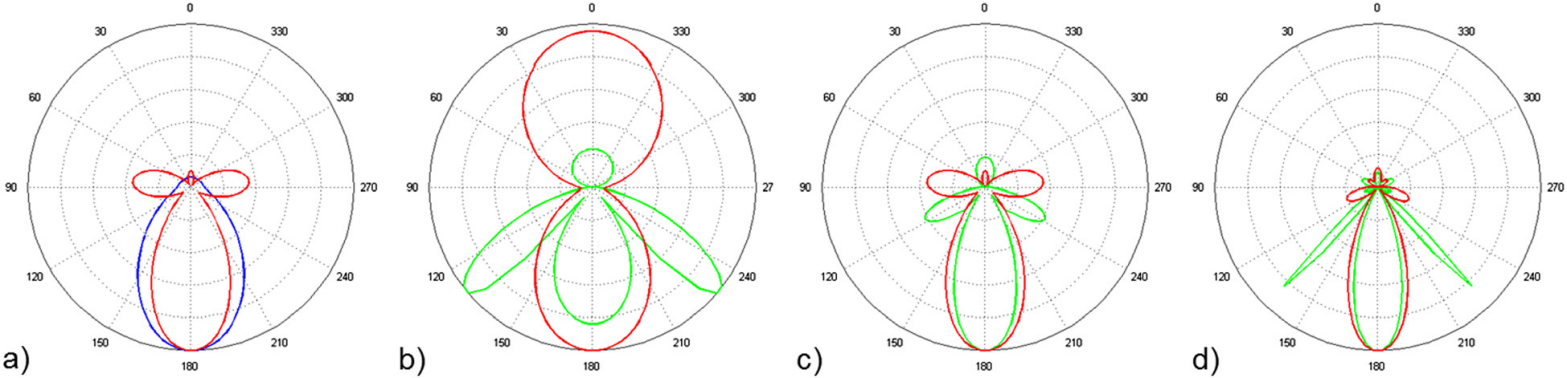
**Angular scattering distributions.** Of **(a)** the quadrupole (magnetic) mode at *λ* = 502 nm of a dielectric nanoparticle (*n* = 2, *k* = 0, *r* = 170 nm, in blue) and the quadrupole (electric) mode at *λ* = 426 nm of a metallic nanoparticle (Ag fitted with Drude model, *r* = 120 nm, in red) in air; **(b)** dipole, **(c)** quadrupole, and **(d)** hexapole electric mode of the above mentioned metallic particle in air (red) and on a substrate with *n* = 1.5 (green) at the resonance wavelengths of 688/914 nm **(b)**, 426/524 nm **(c)**, and 340/420 nm **(d)** (incident light from the top).

Figure 7a compares the angular distribution of scattered light for a metallic (Ag Drude fit) to that of a dielectric (*n* = 2, *k* = 0) nanoparticle (in air) at the respective resonance wavelength of the quadrupole electric or magnetic mode: *λ* = 426 nm for the metallic nanoparticle with 120 nm radius and *λ* = 502 nm for the dielectric one with *r* = 170 nm. For the dielectric nanoparticle, the forward scattering dominates whereas for the metallic nanoparticle, additional lobes emerge, which for the higher order modes, are additionally directed sidewards.

Up to now, we were investigating the nanoparticles in a homogeneous surrounding of *n* = 1 (i.e., in vacuum/air). With respect to the application in a device, placing the nanoparticles at an interface is a more realistic configuration. This also plays an important role when judging about scattering efficiencies. In the following, we will consider the case of a spherical nanoparticle embedded 50 % into a substrate. This symmetric configuration is readily comparable to the situation of a nanoparticle in a homogeneous medium, and there is a comparable experimental configuration where the nanoparticle is embedded into a rough front side layer of the device. The following simulations of nanoparticles at interfaces rely on full 3D simulations as they are performed with the finite element method because Mie theory is not capable of taking substrates into account.

Firstly, the integration of the nanoparticle into a substrate leads to a well-known redshift of the plasmonic resonances. For the Ag nanoparticle with the dielectric function fitted to the Drude model and a radius of 120 nm, the dipole resonance shifts from 688 to 914 nm when embedding it into a substrate with refractive index *n* = 1.5. But secondly, and here most importantly, the angular distribution of the scattered light experiences a stronger orientation to the forward direction and additional sidewards pointing lobes become more pronounced. Figure 7b,c,d highlights this observation by comparing the scattering distribution of the dipole, the quadrupole, and the hexapole mode in air and on the substrate at the respective resonance wavelengths.

**Figure 8:**
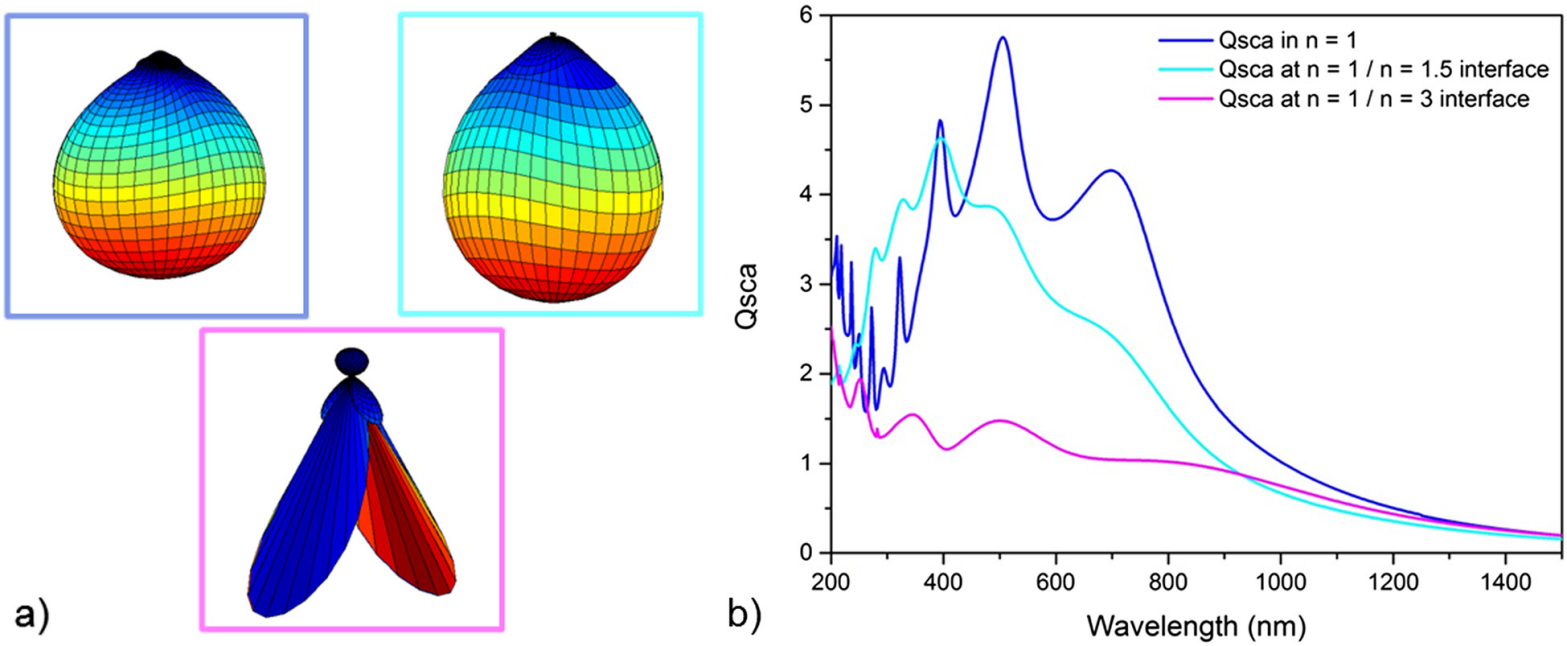
**Angular scattering distribution and scattering cross section for a dielectric nanoparticle at an interface. (a)** Angular distribution of light scattered from an *r* = 170 nm, *n* = 2, k = 0 dielectric nanoparticle in air, i.e., *n* = 1 (blue), at an air/*n* = 1.5 interface (turquoise) and at an air/*n* = 3 interface (magenta) (incident light from the top); **(b)** shows the according scattering cross sections from which the wavelengths of the quadrupole resonance were chosen for the representation of the angular distributions in **(a)**, i.e., 502, 490, and 502 nm.

Thus, in the case of metallic nanoparticles, the embedding into a substrate helps to broaden the angular distribution of the scattered light and to potentially trap it in a thin layer. But how about the dielectric nanoparticles with their initial preferential scattering to the forward direction? Figure 8 represents in subfigure a the 3D angular distribution of the light scattered from an *r* = 170 nm, *n* = 2, *k* = 0 nanoparticle at the resonance of the quadrupole magnetic mode when situated in air (blue legend) and half in air, half in an *n* = 1.5 substrate (turquoise legend). The shape appears almost unchanged, rather reduced to a smaller range of angles when considering that normally, the propagation angles of light will increase inside a substrate due to Snell’s law. Thus, the strong forward scattering remains for this substrate which however has a lower refractive index than the nanoparticle itself. Also, the scattering cross section becomes narrowed and the resonance peaks even blueshifted, see Figure 8b. In contrast, the substrate refractive index was set to *n* = 3 for the third angular scattering distribution shown in Figure 8a (magenta legend). Now that the substrate refractive index is larger than the particle refractive index, a strongly pronounced scattering into higher angle modes is observed. Therefore, it appears that also dielectric nanoparticles can profit from an enhanced angular distribution of scattered light when embedded into a high refractive index substrate. Yet, the normalized scattering cross section is not just redshifted but also subject to a strong damping, see Figure 8b. This damping is significantly more pronounced than for metallic nanoparticles - more than 60 % here compared to approximately 20 % in the corresponding case of metals (see also Additional file 4: Figure S4).

**Figure 9:**
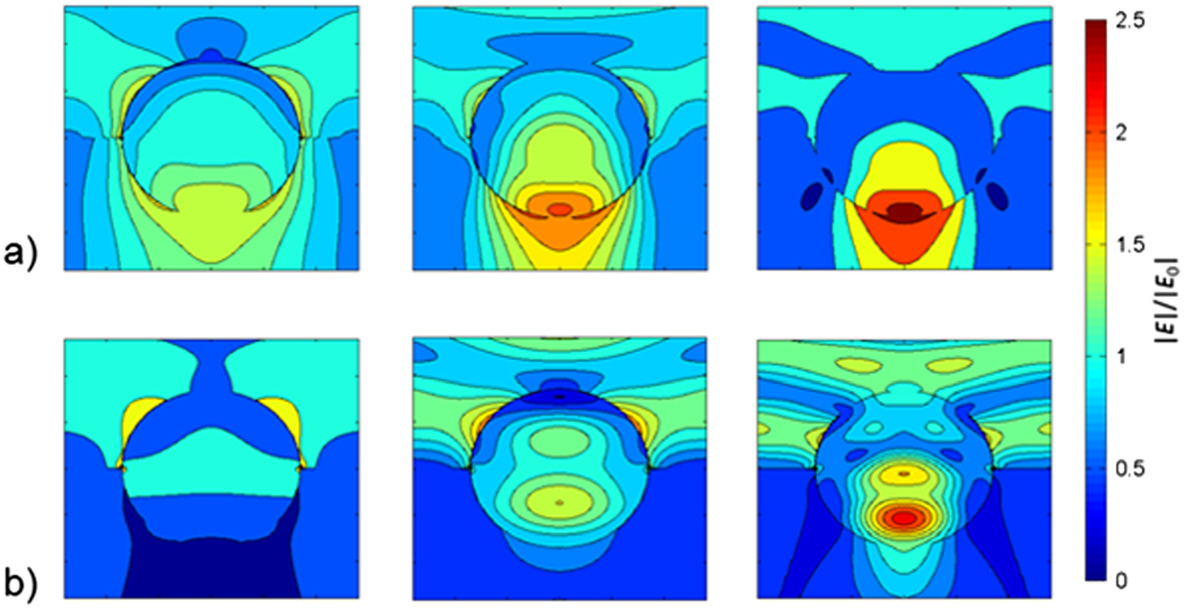
**Near field distributions of a dielectric nanoparticle at an interface.** Electromagnetic field around a dielectric nanoparticle *n* = 2, *k* = 0, and *r* = 170 nm, embedded half in air, half in a substrate with refractive index **(a)***n* = 1.5 and **(b)***n* = 3. The dipole, the quadrupole, and the hexapole modes are shown for the wavelengths of 680/816 nm, 490/502 nm, and 396/346 nm, respectively, which correspond to the maxima in scattering, see Figure 8b (incident light from the top).

Finally, with the integration of a substrate, leaky modes may emerge for the dielectric nanoparticles that, like enhanced near fields, can promote absorption in the underlying layer. Figure 9 shows the electromagnetic near field distribution around the dielectric nanoparticle with *n* = 2, *k* = 0, and *r* = 170 nm when embedded half in air and half in the substrate with (subfigure a) *n* = 1.5 and (subfigure b) *n* = 3. For the case of the low-index substrate, we find stronger forward scattering, which is in agreement with the angular scattering distributions, and the local field in the direct forward direction is enhanced and appears more pronounced than for the nanoparticle in air, compare Figure 4b. However, for the high-index substrate, the local electromagnetic field is more concentrated inside the nanoparticle or directed sidewards which can be correlated to the angular scattering distribution as well. Seeing these two cases together, we can conclude that leaky modes from dielectric nanoparticles occur if the substrate refractive index is lower than the one of the nanoparticles and that the local fields are more pronounced in the material with the lower refractive index (which also may be the nanoparticle if the substrate has a higher refractive index).

A high angular scattering distribution is present for metallic nanoparticles in vacuum and can easily be reinforced by the integration of a substrate without showing significant losses in overall scattering efficiency. Yet, for the dielectric nanoparticles, a high angular scattering distribution can only be achieved for high-index substrates which comes along with significant losses in scattering efficiency; these dielectric nanoparticles may rather benefit from leaky modes appearing with low-index substrates that can lead to enhanced absorption similar to the enhanced near fields around metallic nanoparticles.

## Conclusions

Evaluating scattering and near field properties of metallic and dielectric nanoparticles, we firstly found that the scattering cross sections can, in both cases, reach a value of several times the geometrical cross sections. For the dielectric nanoparticles, no parasitic absorption exists, whereas for the metallic ones, non-zero absorption cross sections are present, which however can be reduced by increasing the particle radius. The nanoparticle radius can be used to tune the resonance position to the desired wavelengths. Scattering cross section maps, calculated here with Mie theory, give a fast overview of the parameter field and quickly show that dielectric nanoparticles with a refractive index around 2 require significantly larger radii (approximately 1.5 times) than metallic ones from, e.g., Ag in order to obtain similar resonance wavelengths. The electromagnetic near fields around the two different nanoparticle types also significantly differ; whereas for the metallic nanoparticles, the field vanishes inside and builds up a strong localized field around the surface, the dielectric nanoparticles have strong fields inside, which however are not absorbed but preferentially scattered to the forward direction. These observations of both typical dielectric and metallic near-fields are found for semiconducting materials. On the one hand, they have a region of constant refractive index and zero absorption and thus a dielectric-like scattering behavior, but on the other hand, they can also show significant charge carriers and thus metallic plasmon resonances. However, since the semiconductor also has a band gap and according high absorption for wavelengths below, it may only be of interest when the band to band absorption is outside the wavelength range in focus. Although semiconductors show the scattering properties of both dielectrics and metals, it was not possible to combine the two effects constructively. Depending on the application, one or the other type of material by itself may be preferred to a combination of both.

Aside from the scattering ability and the near field distribution, also the angular distribution of the scattered light plays a crucial role for applications. Considering in particular the application to ultra-thin solar cells, both an enhanced near field and a particular scattering of the nanoparticle may contribute to enhance the absorption. In a homogeneous medium, the near field is stronger around the metallic nanoparticle, the scattering efficiency (scattering over scattering plus absorption) is stronger for non-absorbing dielectric nanoparticles, so that up to that point, no decision about the ideal choice of material can be made. However, when looking at the angular distribution of the scattered light, we saw that the dielectric nanoparticles show a strong scattering in just the forward direction, but the metallic nanoparticles immediately give rise to scattering lobes directed to high angles. This scattering into high angles allows light trapping in thin layers and therefore absorption enhancement. Scattering lobes in directions which are otherwise subject to total reflection work most efficiently and are preferentially characteristic for metallic nanoparticles. Bringing the nanoparticles at an interface of two materials as it is the typical configuration in solar cells, further improves the high angle scattering of metallic nanoparticles. For dielectric nanoparticles, an interface can also give rise to additional lobes directed to high angles, yet this is only the case when the refractive index of the substrate is larger than the one of the nanoparticle. In the end, this means a high-index substrate, which however causes significant damping to the scattering efficiency for dielectric nanoparticles. The low-index substrate in combination with dielectric nanoparticles may be beneficial for generating leaky modes with a strong near field directed towards the substrate that can lead to absorption enhancement. So after the trade-off between high scattering efficiency and pronounced scattering into high angles, between enhanced near fields and leaky modes, there is no ultimate preferential choice between metallic and dielectric nanoparticles. Yet, Table 2 gives an overview on the main aims for efficient scattering and near fields and how they may be fulfilled, having in mind the example of absorption enhancement in solar cells. For the final evaluation of performance of a certain nanoparticle integrated in e.g., a solar cell, the complete device structure including the nanoparticles and the specific geometry then has to be calculated. It will also depend on the particular solar cell concept whether near field enhancement or scattering turns out more beneficial [28,29]. Finally, aside the theoretical optimization, experimental boundary conditions will define the configurations that are feasible in the end.

**Table 2:** Aims and according requirements for efficient nanoparticle scattering and near fields with the special background of application in solar cells

**Aims**	**Metallic nanoparticles**	**Dielectric nanoparticles**	**Semiconductor nanoparticle**
High scattering efficiency (low absorption)	For big particles	**✓**	Dielectric or metal like behavior depending on the according properties dominating the dispersion
High near-field enhancement	**✓**	Improved at interface (see leaky modes below)	Dielectric or metal like behavior depending on the according properties dominating the dispersion
High scattering into the solar cell	✓	**✓**Scattering towards higher n	Dielectric or metal like behavior depending on the according properties dominating the dispersion
High scattering into large angles	✓	✓On high-index substrate	Dielectric or metal like behavior depending on the according properties dominating the dispersion
High scattering efficiency at interface	✓	Drop in scattering efficiency	Dielectric or metal like behavior depending on the according properties dominating the dispersion
High local leaky modes	✓	✓On low-index substrate	Dielectric or metal like behavior depending on the according properties dominating the dispersion

## Competing interests

The authors declare that they have no competing interests.

## Authors’ contributions

MS developed the idea of comparing optical scattering and near field properties of nanoparticles made from different materials. She drafted the manuscript and ran the simulations. PA provided and adapted the code for the Mie simulations and PM set up the FEM calculations. All authors contributed to the preparation and revision of the manuscript. All authors read and approved the manuscript.

## Authors’ information

MS is the leader of the Young Investigator Group ‘Nanooptical concepts for Chalcopyrite solar cells’ at the Helmholtz-Zentrum Berlin. PA and PM are PhD students in the group.

## Supplementary Material

Additional file 1: Figure S1 Absorption cross section of a 120-nm radius Ag nanoparticle with dielectric function according to a Drude fit: sum and allocation to different modes. Click here for file

Additional file 2: Figure S2 Map of scattering cross section for a spherical dielectric nanoparticle with *n* = 2 and *k* = 0. Click here for file

Additional file 3: Figure S3 Maps of (a) scattering cross section and (b) scattering efficiency for a spherical nanoparticle from GZO semiconductor (refractive index data fitted with parameters from [27]). Click here for file

Additional file 4: Figure S4 Scattering cross section of a Ag nanoparticle (fitted with Drude model) of *r* =120 nm in vacuum and when placed onto a substrate with *n* = 1.5. Click here for file
