# Supplementary figures and images for: Plasmonic and photonic scattering and near fields of nanoparticles
Source: Nanoscale Res Lett. 2014 Jan 29;9(1):50. doi: 10.1186/1556-276X-9-50 (PMC3915561; doi:10.1186/1556-276X-9-50)

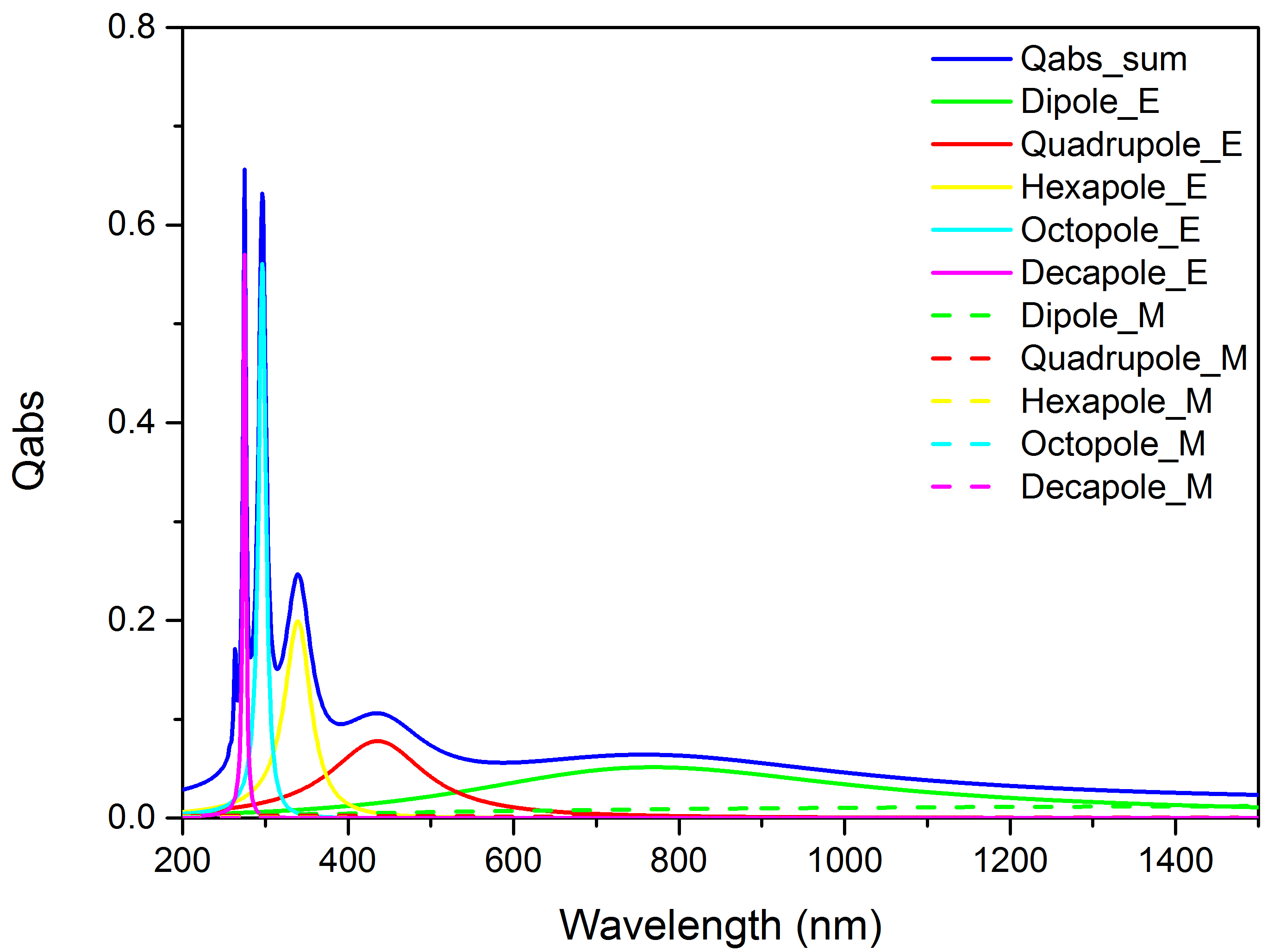

Supplement: Additional file 1: Figure S1 — Absorption cross section of a 120-nm radius Ag nanoparticle with dielectric function according to a Drude fit: sum and allocation to different modes. [file 1556-276X-9-50-S1.jpeg]

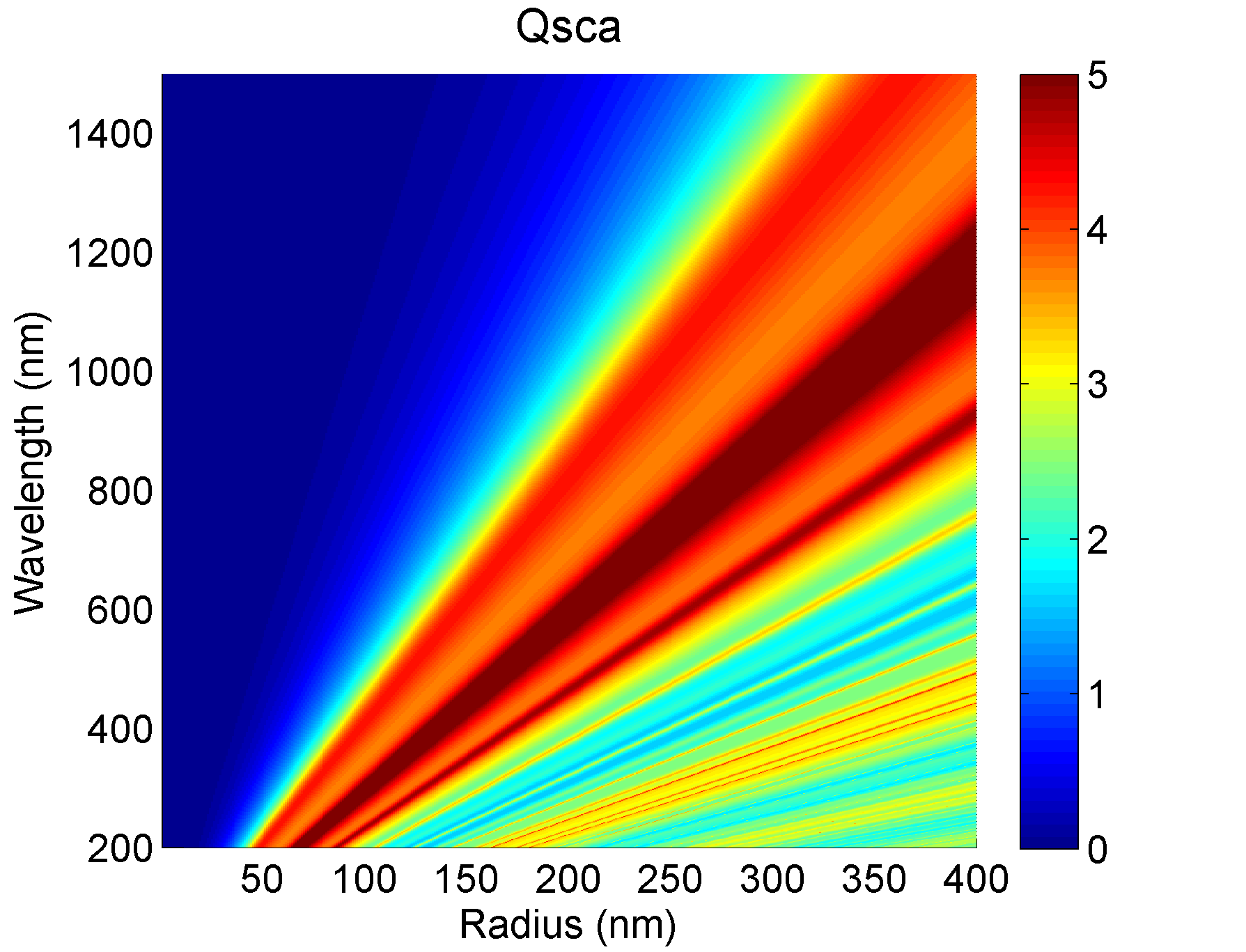

Supplement: Additional file 2: Figure S2 — Map of scattering cross section for a spherical dielectric nanoparticle with n = 2 and k = 0. [file 1556-276X-9-50-S2.png]

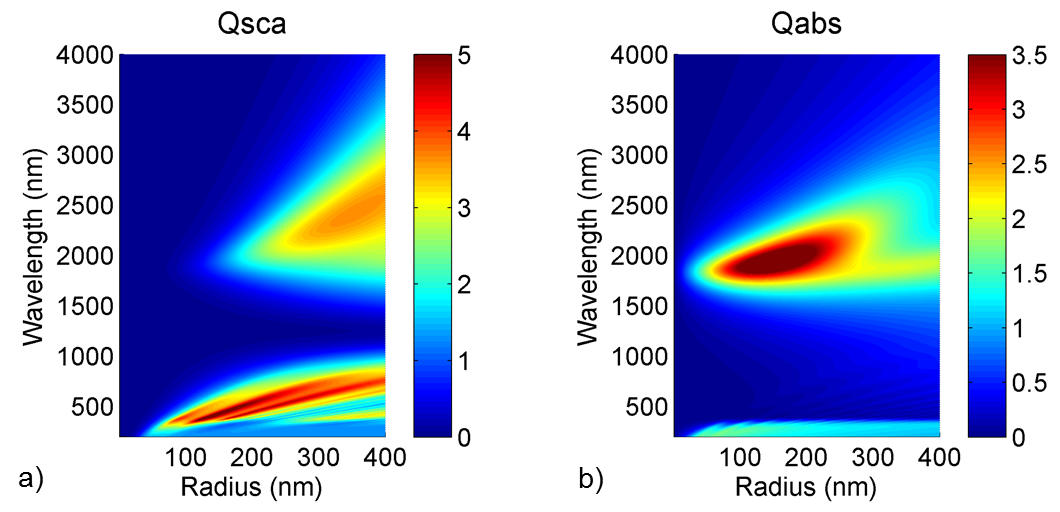

Supplement: Additional file 3: Figure S3 — Maps of (a) scattering cross section and (b) scattering efficiency for a spherical nanoparticle from GZO semiconductor (refractive index data fitted with parameters from [27]). [file 1556-276X-9-50-S3.tiff]

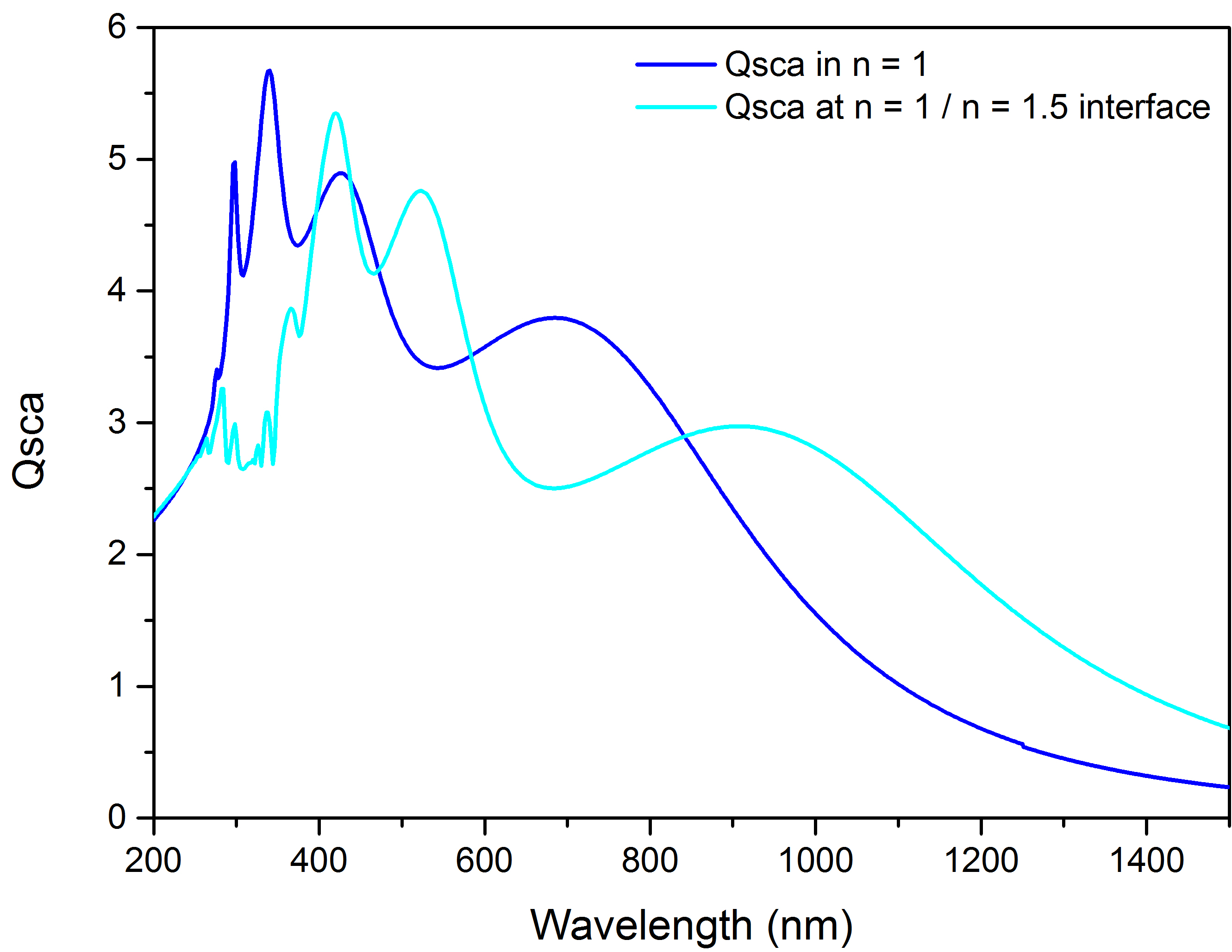

Supplement: Additional file 4: Figure S4 — Scattering cross section of a Ag nanoparticle (fitted with Drude model) of r =120 nm in vacuum and when placed onto a substrate with n = 1.5. [file 1556-276X-9-50-S4.jpeg]
